# Genome Sequences of Nine Erwinia amylovora Bacteriophages

**DOI:** 10.1128/MRA.00944-18

**Published:** 2018-10-11

**Authors:** Ruchira Sharma, Jordan A. Berg, Nolan J. Beatty, Minsey C. Choi, Ashlin E. Cowger, Brooke J. R. Cozzens, Steven G. Duncan, Christopher P. Fajardo, Hannah P. Ferguson, Trevon Galbraith, Jacob A. Herring, Taalin R. Hoj, Jill L. Durrant, Jonathan R. Hyde, Garrett L. Jensen, Si Yang Ke, Shalee Killpack, Jared L. Kruger, Eliza E. K. Lawrence, Ifeanyichukwu O. Nwosu, Tsz Ching Tam, Daniel W. Thompson, Josie A. Tueller, Megan E. H. Ward, Charles J. Webb, Madison E. Wood, Edward L. Yeates, David A. Baltrus, Donald P. Breakwell, Sandra Hope, Julianne H. Grose

**Affiliations:** a Microbiology and Molecular Biology Department, Brigham Young University, Provo, Utah, USA; b School of Plant Sciences, The University of Arizona, Tempe, Arizona, USA; Louisiana State University

## Abstract

Erwinia amylovora is a plant pathogen belonging to the Enterobacteriaceae family, a family containing many plant and animal pathogens. Herein, we announce nine genome sequences of E. amylovora bacteriophages isolated from infected apple trees along the Wasatch Front in Utah.

## ANNOUNCEMENT

At an estimated total number of 10^31^, phages are by far the most abundant biological entity on the planet (1–7). They dramatically influence the evolution of bacteria by their ability to infect and kill their hosts and to transfer genetic material. Erwinia amylovora is a rod-shaped facultative anaerobic member of the Enterobacteriaceae bacterial family, which includes many well-characterized Gram-negative plant and animal pathogens, such as Salmonella spp., Escherichia coli, and Klebsiella spp. As the causative agent of fire blight, Erwinia amylovora infects members of the Rosaceae plant family, causing diseased areas to appear burnt (8–10). The isolation and characterization of phages that infect E. amylovora may aid in our understanding of these bacteria and provide potential treatment for this devastating agricultural disease. Herein, we announce the genome sequences of nine E. amylovora bacteriophages, vB_EamM_Asesino, vB_EamM_Alexandra, vB_EamM_Bosolaphorus, vB_EamM_Desertfox, vB_EamM_MadMel, vB_EamM_Mortimer, vB_EamP_Pavtok, vB_EamM_SunLIRen, and vB_EamM_Wellington.

Phages were isolated from apple trees along the Wasatch Front in Utah that appeared to harbor fire blight infection. Phages were plaque purified through a minimum of three passages after amplification via enrichment culture (11). All nine phages reported in this announcement infect the Erwinia amylovora ATCC 29780 strain, as indicated by plaque assays, and their characteristics are summarized in Table 1. Genomic DNA was extracted (Phage DNA isolation kit; Norgen Biotek), a library was made using the Illumina TruSeq DNA Nano kit, and sample genomes were sequenced by Illumina HiSeq 2500 sequencing (250-bp paired end) and assembled with Geneious (12) version 8.1 using *de novo* assembly with medium-low sensitivity and various percentages of data. All phages circularized upon assembly and were annotated using DNA Master (http://cobamide2.bio.pitt.edu/computer.htm), giving preference for calls that gave full coding potential coverage.

**TABLE 1:** Properties of nine *Erwinia amylovora* bacteriophage genomes

Name	GenBank accession no.	SRA accession no.	Total no. of reads	No. of reads used	Assembly fold coverage (range [mean])	Length (bp)	No. of ORFs^*a*^	No. of tRNAs	G+C content (%)
vB_EamP_Pavtok	MH426726	SRX4597602	1,301,332	386,192	492–2,086 (1,069)	61,401	62	0	36.9
vB_EamM_SunLIRen	MH426725	SRX4597606	1,301,332	386,192	8,249–42,422 (13,566)	84,559	141	22	36.3
vB_EamM_Wellington	MH426724	SRX4597603	626,048	372,488	133–514 (329.7)	244,950	295	8	50.3
vB_EamM_Asesino	KX397364	SRX4597609	2,222,038	1,022,382	512–1,378 (1,037.7)	246,290	289	12	51.2
vB_EamM_Alexandra	MH248138	SRX4597608	381,540	200,005	63–516 (166.3)	266,532	349	0	49.8
vB_EamM_Bosolaphorus	MG655267	SRX4597604	778,168	326,344	83–555 (248.4)	272,228	321	1	49.4
vB_EamM_Desertfox	MG655268	SRX4597605	1,930,470	1,138,933	115–612 (352.9)	272,458	320	0	49.6
vB_EamM_Mortimer	MG655270	SRX4616109	2,581,160	287,396	47–207 (129.4)	273,914	325	1	49.5
vB_EamM_MadMel	MG655269	SRX4597607	1,604,720	1,443,568	567–1,577 (1,213.9)	275,000	321	0	49.4

a ORFs, open reading frames based on current annotation.

The nine phages were grouped into five distinct clusters by genomic dot plot and average nucleotide identity analyses, as previously described (11), with the first three groups containing jumbo Myoviridae. The first jumbo group included four myoviruses, vB_EamM_Bosolaphorus, vB_EamM_Desertfox, vB_EamM_MadMel, and vB_EamM_Mortimer, which are similar to previously published Erwinia phage Ea35-70 (13), as well as other phages we have isolated (14). The second group included two jumbo myoviruses, vB_EamM_Asesino and vB_EamM_Wellington, with similarity to the well-characterized Salmonella SPN3US phage (15) and related phages. The third is a single jumbo myovirus, EamM_Alexandra, which has similarity to previously published Erwinia phages EamM_Yoloswag (14) and EamM_Y3 (16). Podovirus vB_EamP_Pavtok and myovirus vB_EamM_SunLIRen are similar to Erwinia phages PEp14 and phiEa21-4 (17), respectively. The three jumbo myovirus groups package DNA by headful packaging (14) based on homology to phage phiKZ terminase (18), and their bp 1 was chosen by alignment to their phage family. PhageTerm (19) was used to determine the packaging strategy of SunLIRen and Pavtok. SunLIRen appeared to have headful packaging, and its bp 1 was assigned based on homology alignment to Erwinia phage phiEa21-4, while the packaging strategy of Pavtok is unknown, and its bp 1 was assigned due to homology to PEp14.

### Data availability.

The GenBank and SRA accession numbers for the nine Erwinia bacteriophages are listed in Table 1.

## References

[1] Bergh O, Børsheim KY, Bratbak G, Heldal M 1989 High abundance of viruses found in aquatic environments. Nature 340:467–468. doi:10.1038/340467a0. 2755508

[2] Wommack KE, Colwell RR 2000 Virioplankton: viruses in aquatic ecosystems. Microbiol Mol Biol Rev 64:69–114. doi:10.1128/MMBR.64.1.69-114.2000. 10704475 PMC98987

[3] Brüssow H, Hendrix RW 2002 Phage genomics: small is beautiful. Cell 108:13–16. doi:10.1016/S0092-8674(01)00637-7. 11792317

[4] Wilhelm SW, Jeffrey WH, Suttle CA, Mitchell DL 2002 Estimation of biologically damaging UV levels in marine surface waters with DNA and viral dosimeters. Photochem Photobiol 76:268–273. 12403447 10.1562/0031-8655(2002)076<0268:eobdul>2.0.co;2

[5] Hendrix RW 2003 Bacteriophage genomics. Curr Opin Microbiol 6:506–511. doi:10.1016/j.mib.2003.09.004. 14572544

[6] Hambly E, Suttle CA 2005 The viriosphere, diversity, and genetic exchange within phage communities. Curr Opin Microbiol 8:444–450. doi:10.1016/j.mib.2005.06.005. 15979387

[7] Suttle CA 2005 Viruses in the sea. Nature 437:356–361. doi:10.1038/nature04160. 16163346

[8] Khan MA, Zhao Y, Korban SS 2012 Molecular mechanisms of pathogenesis and resistance to the bacterial pathogen *Erwinia amylovora*, causal agent of fire blight disease in rosaceae. Plant Mol Biol Rep 30:247–260. doi:10.1007/s11105-011-0334-1.

[9] Schroth M, Thomson S, Hildebrand D, Moller W 1974 Epidemiology and control of fire blight. Annu Rev Phytopathol 12:389–412. doi:10.1146/annurev.py.12.090174.002133.

[10] Thomson SV 2000 Epidemiology of fire blight 2, p 9–36. *In* Vanneste J (ed), Fire blight: the disease and its causative agent, *Erwinia amylovora*. CABI Publishing, Wallingford, United Kingdom.

[11] Arens DK, Brady TS, Carter JL, Pape JA, Robinson DM, Russell KA, Staley LA, Stettler JM, Tateoka OB, Townsend MH, Whitley KV, Wienclaw TM, Williamson TL, Johnson SM, Grose JH 2018 Characterization of two related *Erwinia* myoviruses that are distant relatives of the PhiKZ-like jumbo phages. PLoS One 13:e0200202. doi:10.1371/journal.pone.0200202. 29979759 PMC6034870

[12] Kearse M, Moir R, Wilson A, Stones-Havas S, Cheung M, Sturrock S, Buxton S, Cooper A, Markowitz S, Duran C, Thierer T, Ashton B, Meintjes P, Drummond A 2012 Geneious Basic: an integrated and extendable desktop software platform for the organization and analysis of sequence data. Bioinformatics 28:1647–1649. doi:10.1093/bioinformatics/bts199. 22543367 PMC3371832

[13] Yagubi AI, Castle AJ, Kropinski AM, Banks TW, Svircev AM 2014 Complete genome sequence of Erwinia *amylovora bacteriophage* vB_EamM_Ea35-70. Genome Announc 2:e00413-14. doi:10.1128/genomeA.00413-14. 25146132 PMC4153477

[14] Esplin IND, Berg JA, Sharma R, Allen RC, Arens DK, Ashcroft CR, Bairett SR, Beatty NJ, Bickmore M, Bloomfield TJ, Brady TS, Bybee RN, Carter JL, Choi MC, Duncan S, Fajardo CP, Foy BB, Fuhriman DA, Gibby PD, Grossarth SE, Harbaugh K, Harris N, Hilton JA, Hurst E, Hyde JR, Ingersoll K, Jacobson CM, James BD, Jarvis TM, Jaen-Anieves D, Jensen GL, Knabe BK, Kruger JL, Merrill BD, Pape JA, Payne Anderson AM, Payne DE, Peck MD, Pollock SV, Putnam MJ, Ransom EK, Ririe DB, Robinson DM, Rogers SL, Russell KA, Schoenhals JE, Shurtleff CA, Simister AR, Smith HG, Stephenson MB, et al 2017 Genome sequences of 19 novel *Erwinia amylovora* bacteriophages. Genome Announc 5:e00931-17. doi:10.1128/genomeA.00931-17. 29146842 PMC5690319

[15] Lee JH, Shin H, Kim H, Ryu S 2011 Complete genome sequence of *Salmonella* bacteriophage SPN3US. J Virol 85:13470–13471. doi:10.1128/JVI.06344-11. 22106383 PMC3233126

[16] Buttimer C, Born Y, Lucid A, Loessner MJ, Fieseler L, Coffey A 2018 *Erwinia amylovora* phage vB_EamM_Y3 represents another lineage of hairy *Myoviridae*. Res Microbiol 30062–30067. doi:10.1016/j.resmic.2018.04.006. 29777834

[17] Lehman SM, Kropinski AM, Castle AJ, Svircev AM 2009 Complete genome of the broad-host-range *Erwinia amylovora* phage phiEa21-4 and its relationship to *Salmonella* phage Felix O1. Appl Environ Microbiol 75:2139–2147. doi:10.1128/AEM.02352-08. 19181832 PMC2663227

[18] Mesyanzhinov VV, Robben J, Grymonprez B, Kostyuchenko VA, Bourkaltseva MV, Sykilinda NN, Krylov VN, Volckaert G 2002 The genome of bacteriophage phiKZ of *Pseudomonas aeruginosa*. J Mol Biol 317:1–19. doi:10.1006/jmbi.2001.5396. 11916376

[19] Garneau JR, Depardieu F, Fortier L-C, Bikard D, Monot M 2017 PhageTerm: a fast and user-friendly software to determine bacteriophage termini and packaging mode using randomly fragmented NGS data. Sci Rep 7:8292. doi:10.1038/s41598-017-07910-5. 28811656 PMC5557969

